# Bystander Intervention in Coercive Control: Do Relationship to the Victim, Bystander Gender, and Concerns Influence Willingness to Intervene?

**DOI:** 10.1177/08862605241234350

**Published:** 2024-02-26

**Authors:** Jacqueline Walker, Sally Fiona Kelty, Caroline Ng Tseung-Wong

**Affiliations:** 1 University of Canberra, ACT, Australia

**Keywords:** coercive control, bystander intervention concerns, intimate partner violence, friendship and bystander intervention, intervention programs

## Abstract

With rates of coercive control (CC) increasing, there is a need to ensure that intervention programs are underpinned by evidence-based research. Current interventions are scarce, with their efficacy rarely established. Most current interventions appear to rely on victims seeking support from formal sources/agencies, despite suggestions that victims are more likely to confide in people they know, such as their friends. Researchers suggest that a victim’s friends may provide an effective source of support and intervention. The aim of this study was to fill the gap in the literature exploring whether the closeness of the relationship to the victim, bystander gender, and bystander concerns influenced attitudes toward intervening in CC situations. The study used an experimental design, whereby participants were randomly allocated to read a vignette depicting a CC scenario involving a friend, colleague, or stranger, and quantitative methods were used to examine bystanders’ willingness and concerns about intervening. The sample was 340 Australian participants (229 female, 111 male), recruited from social media, namely community Facebook groups. The results indicated that friends were significantly more willing to intervene than colleagues or strangers, while strangers reported the highest concerns about intervening. Females reported significantly higher willingness to intervene than men despite also reporting higher concerns. Exploratory analysis of concerns about intervening revealed that the participants were most concerned about risk of harm and their beliefs in their ability to successfully intervene. These findings have implications for bystander intervention programs and campaigns, including offering a range of potential directions to enhance intervention program content.

Intimate partner violence (IPV) is defined as any form of physical, psychological, or sexual violence carried out between current or former intimate partners (Bohall et al., 2016). Both men and women can perpetrate IPV; however, research suggests that women are significantly more likely to experience IPV victimization (Tanha et al., 2009). For example, in Australia, approximately 1 in 6 women and 1 in 16 men aged 15 and above will experience IPV in their lifetime (Australian Bureau of Statistics, 2017).

Recent media attention has raised awareness that a major component of IPV is non-physical abuse, known as coercive control (CC). CC is a pattern of behavior used by one partner to dominate another through non-physical tactics, such as isolation, economic abuse, technological monitoring, and emotional abuse (Dichter et al., 2018). Each of these tactics is used to control the victim and can have extreme consequences for victims’ mental health and self-esteem (Hegarty et al, 2020; Lutwak, 2018). While CC behaviors do not involve physical violence, researchers have identified strong correlations between CC and physical abuse, including its role in predicting intimate partner homicide (Graham-Kevan & Archer, 2008; Monckton Smith, 2020).

As CC commonly occurs in the early stages of many intimate relationships that become physically violent (Graham-Kevan & Archer, 2008), researchers suggest that interventions targeting CC may be effective in stopping the progression to physical aggression (West & Wandrei, 2002). However, many current interventions targeting IPV/CC appear to be ineffective (Bohall et al., 2016; Herman et al., 2014; Stark & Hester, 2018). For example, perpetrator programs have high attrition rates and low efficacy in reducing recidivism (Dutton & Corvo, 2007; Gondolf, 2000). Legislation has been implemented in some countries, including the United Kingdom, France, and some Australian jurisdictions (Stark & Hester, 2018). While legislation can impose legal consequences for perpetrators of CC, issues with reporting and policing of CC have raised concerns regarding its efficacy as an intervention (Hardesty et al., 2015). Moreover, legislation relies on victims to formally report abuse despite there being a range of barriers that prevent this from occurring (Hamberger et al., 2017; Walklate & Fitz-Gibbon, 2019). Therefore, there is a growing demand for more informal interventions to combat rising rates of CC and IPV.

## The Effect of Friendship on Bystander Intervention

Most research exploring bystander intervention in IPV is underpinned by Latané and Darley’s (1970) bystander intervention model (BIM). This model proposes five steps in intervening: (1) notice the event, (2) interpret as an emergency, (3) assume responsibility, (4) plan the intervention, and (5) implement the intervention. Each step incorporates a range of cognitive processes or decisions that either promote or demote bystander intervention.

Studies of helping suggest people are more likely to move through the BIM steps when the victim is a friend (Chabot et al., 2018; Stavrou et al., 2016; Taylor et al., 2019). For example, Graziano et al. (2007) examined the helping behaviors of 622 United States (U.S.) undergraduate students. They found the relationship with the victim moderated the likelihood of engaging in helping behaviors, whereby participants were significantly more likely to help family and friends than strangers (Graziano et al., 2007). Similar results have been found across a range of situations showing bystanders are more likely to help a “familiar stranger” than a complete stranger (Milgram, 1977; Pearce, 1980). This effect may occur due to reciprocal norms underpinning friendships (Argyle & Henderson, 1984). The following sections will explore the effect of friendship on each of the BIM steps.

### Notice the Event

CC behaviors can be subtle, making them hard to identify, especially in the context of a relationship that is unknown to the bystander (Boxall & Morgan, 2021). However, research on help-seeking suggests that victims prefer to confide in friends over strangers. In an Australian sample (*N* = 469), Stavrou et al. (2016) found that of the 69% of IPV victims who reported seeking help, 76% had initially confided in a friend. Other studies have replicated similar results (e.g., Fanslow & Robinson, 2009; Hanson et al. 2003), thus suggesting that friends of victims may be better placed than others to notice CC in an intimate relationship.

### Interpret the Event as an Emergency

As CC relationships lack physical violence, the severity of the abuse may be unclear to bystanders (Stark & Hester, 2018). It is possible, though, that friends may be more likely to observe the effects of CC on the victim (i.e., mental and physical health issues) (Fanslow & Robinson, 2009). In a sample of 545 U.S. undergraduate students, Bennett and Banyard (2016) found that friends of sexual assault (SA) victims’ were more likely to state that SA was highly problematic than strangers. These results suggest that in perceiving the event as more severe, friends may be more likely to interpret it as an emergency situation that warrants intervention.

### Assume Intervention Responsibility

Research on attribution theory suggests that friends, more than strangers, will take personal responsibility for an intervention (Chabot et al., 2009). A study by Palmer et al. (2018) explored the effect of friendship on the attributions of responsibility in hypothetical IPV and SA scenarios using 721 U.S. students. Results showed those who knew the victim were significantly more likely to intervene, either directly or indirectly (i.e., confront the perpetrator or create a distraction). On the contrary, strangers tended to ignore or delegate the intervention responsibility (i.e., call the police) (Palmer et al., 2018).

### Plan the Intervention

Research suggests the probability of intervention increases when bystanders are confident that they can intervene successfully (Latané & Darley, 1970). Friends of victims appear to report more confidence in their ability to intervene, potentially due to their knowledge and awareness of the situation and personal relationship with the victim (Bannon & Foubert, 2017; Krieger et al., 2017). However, research investigating the effects of friendship and confidence on bystander interventions in CC appears to be lacking.

## Bystander Calculus Model: Concerns About Intervening

Friendship may also influence the perceived concerns (i.e., costs) and rewards of intervening, impacting the overall likelihood of engaging in an intervention (Levine et al., 2005). This effect was explained in Piliavin et al.’s (1981) bystander calculus model, which suggests that bystanders subconsciously conduct a cost-reward analysis when deciding whether to intervene in an emergency. To reduce the arousal resulting from an emergency, bystanders consider their concerns and potential rewards of intervening and respond according to the best method of arousal reduction (Dovidio et al., 1991). When the rewards outweigh their concerns, the likelihood of engaging in an intervention increases. For example, Banyard et al. (2021) explored 966 bystanders’ perceived consequences of helping in an SA situation. They found that bystanders reported higher intent to intervene when they perceived the consequences as positive (Banyard et al., 2021).

There is limited knowledge on concerns for bystanders intervening in CC. One study by Weitzman et al. (2020) evaluated six common concerns reported by bystanders in IPV: (1) physical harm to the victim, bystander or property, (2) misinterpretation (of the need for intervention/the intervention being misinterpreted), (3) making things worse, (4) being unsuccessful/no change occurring, (5) it is a private matter and, for those who knew the victim, and (6) the effect on the relationship. They found mixed results regarding the effect of friendship on the concerns of intervening. For example, friends’ exposure to the context of the relationship and increased self-efficacy reduced concerns about being unsuccessful or misinterpreting the situation (Weitzman et al., 2020). However, the increased concerns about the potential effects on their friendship inhibited intervention likelihood (Weitzman et al., 2020). Other studies have examined similar concerns in relation to intervening with an IPV perpetrator (Mead & Kelty, 2021); however, further exploration is required to better understand the concerns of individuals intervening in CC.

## Gender and Bystander Interventions

Research has evaluated gender differences in tolerance for violence and consequences for bystander interventions (Archer & Haigh, 1997; Campbell & Muncer, 1987). Tolerance for violence is suggested to influence bystander intervention, with research finding the lower the tolerance, the more likely one is to intervene (Smith et al., 2019). As males are more likely to perpetrate IPV (Australian Institute of Health and Welfare [AIHW], 2022), they may have higher tolerance for CC, thus reducing the likelihood of intervening (Smith et al., 2019; Yule & Grych, 2020). In contrast, as females are at greater risk of victimization and appear to be less tolerant of violence, it is suggested this can increase their perceived similarity to CC victims (AIHW, 2022), which may increase the empathy they feel and promote the intervention likelihood (Fourie et al., 2017).

Other research suggests that females experience increased perceived concerns about intervening. Eagly and Steffen’s (1986) meta-analysis of 63 studies found that females may become passive bystanders when the risk of physical harm or consequences to the victim is probable. Thus, females may become reluctant to intervene in potentially physical situations (Eagly & Steffen, 1986). Despite these findings, little is known about the effect of gender on the concerns about intervening in CC, and as the risk of physical violence in CC is less clear, more specific research to CC is required.

## The Current Study

This study aimed to explore whether relationship between the victim and gender influences bystander intervention in CC. Past research on victim help-seeking and bystander intervention in IPV and SA suggested that friends of victims may be uniquely placed to recognize and facilitate CC interventions. However, CC is relatively new to mainstream literature and researchers have not yet explored the bystander role that friends can play in CC situations. In order to understand bystander responses and direct intervention program content, the potential concerns of intervening must also be examined; however, there appears to be limited knowledge in this area. The current study was designed to address these gaps by using quantitative methods to explore the influence of relationship with the victim and gender on willingness to intervene and concerns in doing so. In this study we test two main hypotheses and one exploratory research question. These are as follows:

*Hypothesis 1*: Friends of the victim will report the highest willingness to intervene, followed by work colleagues; the lowest willingness will be strangers. Gender differences are also expected, whereby females will report more willingness to intervene than males.
*Hypothesis 2*: The type of relationship to the victim will significantly influence the concerns about intervening, with friends reporting the least concerns, work colleagues reporting some concerns, and strangers reporting the most concerns. Gender will also significantly influence concerns, whereby females will report higher concerns than males.
*Exploratory question 1*: This question was devised to expand the knowledge of the types of concerns that participants had about intervening in CC situations. As this is an exploratory question, no formal hypothesis was set. The rationale for this exploratory question was that to develop effective interventions and promote bystander interventions, it is important to understand the type of concerns that are most highly reported.

## Method

### Participants

The participants were Australian citizens aged 17 years and over. The final sample were 340 participants (229 female, 111 male), aged 17 to 82 (*M* = 42.66 years, *SD* = 15.84, *Sk* = 0.23). Of the final sample, 84.7% identified as Australian; 13.2% identified as Aboriginal/Torres Strait Islander, 2.6% British, 1.8% European, 1.5% New Zealander, 0.9% Indian, 0.6% Asian, 0.3% Middle Eastern, 0.3% Polynesian, and 4.1% indicated “other.” The educational level of the sample was; 30.9% completed an Undergraduate degree, 25% completed a TAFE/apprenticeship, 18.2% completed Year 12, 15.6% completed Masters/PhD studies, 6.2% completed an Honors degree, and 4.4% completed Year 10 or below.

### Measures

Demographics of age, gender, education, ethnicity, and employment status were collected. To explore the effect of relationship to the victim and gender on (i) willingness to intervene and (ii) concerns about intervening, participants were randomly allocated to read one of three short vignettes, which were constructed using true stories of CC. All vignettes depicted the same incident in a gender and age-neutral couple to remove the effect of potential confound variables. In the vignette, the perpetrator used emotional and financial abuse, technological monitoring, and isolation to control their partner (see Appendix A for the full vignette). The vignettes differed only by relationship to the victim (friends, colleagues, or strangers). All participants answered a manipulation check, asking them to identify their relationship to the couple in the story. The answer was dependent on which condition participants were allocated to ensure that they had read and understood the vignette, while also increasing the salience of each condition.

#### Willingness to Intervene Scale

Willingness to intervene was measured using an adapted 13-item Willingness to Intervene Scale (WTIS) (Nickerson et al., 2014). The items asked participants about their attitudes toward the CC behavior depicted in the vignette. Each item was developed to test one of the steps in Latané and Darley’s (1970) BIM. Items were adapted from previous studies that measured the steps as continuous variables (primarily Nickerson et al., 2014) to fit the context of CC and in accordance with the condition (i.e., friends answered questions about their friends while strangers answered questions about Partners A and B). Participants were asked to indicate their level of agreement with each statement on a 5-point Likert-type scale from 1 (*strongly disagree*) to 5 (*strongly agree*), with a midpoint of 3 (*neither agree or disagree*). A mean composite score was created using the scores on the 13 items. Item 1 was recoded so that higher scores demonstrated higher willingness. Reliability analysis indicated that the scale was moderately reliable (Cronbach’s α = .68). However, Item 2 (“I believe there is a problem with women being dominated by their partners in this country”) was removed due to negatively correlating with the scale (all other items correlated positively) (DeVellis & Thorpe, 2021). Reliability analysis indicated that the scale had high internal consistency (Cronbach’s α = .78).

#### Concerns About Intervening

Concerns about intervening were measured using the adapted Costs to Intervening Scale (CTIS). Eight items measured participants’ concerns about intervening in the situation depicted in the vignette. The items were developed using previous studies that assessed the common concerns reported by bystanders in IPV and SA situations (i.e., Mead & Kelty, 2021; Weitzman et al., 2020). These concerns were adapted to fit the context of the vignette and in accordance with the condition. Participants were asked to indicate their level of agreement with each statement on a 5-point Likert-type scale ranging from 1 (*strongly disagree*) to 5 (*strongly agree*) and with a midpoint of 3 (*neither agree or disagree*). A total of 21 participants did not complete the CTIS due to drop out. All other responses were complete. A mean composite score was created using completed responses across the 8 items. Reliability analysis indicated that the CTIS scale had good internal consistency (Cronbach’s α = .71).

### Procedure

The study was reviewed and approved by the University of Canberra ethics committee, number HREC-11613. Participants were recruited via an advertisement in various community Facebook groups across all Australian states. The advertisement provided potential participants with general information about the study, stating that the study aimed to investigate public attitudes about couples’ interactions, including investigating whether people would get involved in public arguments and any concerns about intervening. The true aim (to investigate the effect of relational distance and gender on CC interventions and concerns) was withheld until the end of the survey to avoid priming participants. The study was open to Australian citizens and permanent residents aged 17 and over. All participants were provided with the chance to win a $25 shopping center voucher.

Once consent was obtained, participants completed demographic questions. Individuals who were under 17 years of age, or were not Australian citizens/permanent residents were redirected to the end of the survey. All other participants were then randomly allocated to a vignette (friend, colleague or stranger condition). Participants were asked to read the vignette and were unable to click “next” for 20 seconds to encourage reading the vignette entirely. Participants then completed a manipulation check to ensure they understood the vignette. Then, participants progressed to the WTIS and CTIS. Upon completion, participants were fully debriefed and given the option to enter the draw to win one of the gift vouchers. The draw information was separate from other responses to ensure confidentiality.

## Results

### Data Screening

From the original dataset (*N* *=* 551), 177 responses were removed due to less than 88% completion (at least one independent variable), failure to provide consent, or not being an Australian citizen/permanent resident. Two cases were removed due to inappropriate and disingenuous responses. Responses were also removed for failing the manipulation check by identifying the incorrect relationship to the victim (friend *n* = 11, colleague *n* = 11, stranger *n* = 5). Five participants identified as LGBTIQ+; however, they were excluded from the analyses due to the extremely small sample size that did not satisfy equal variance (Pallant, 2020). Descriptive statistics were checked for missing data. None were identified in the WTIS, and 21 missing cases were identified in the CTIS. Upon inspection, it appeared that these participants dropped out prior to completion of the survey. They were retained for the analysis of the WTIS but excluded from the CTIS analysis. No extreme univariate or multivariate outliers were identified in either of the analyses. The final sample consisted of 340 adults. The descriptive statistics for each of the IV’s can be found in Table 1. While the sample size was large and indicators of normality appeared satisfactory, a cautious, critical alpha level of .01 was used to reduce the risk of type one error (Tabachnick & Fidell, 2013).

**Table 1.** Descriptive Statistics, Including Number of Participants, Means, and Standard Deviations for Willingness to Intervene and Concerns About Intervening Across Condition and Gender.

Variable	Condition			Gender	
	Friend	Colleague	Stranger	Female	Male
Willingness to intervene (*N*^a^ = 340)	4.06 (0.47)	3.79 (0.51)	3.42 (0.60)	3.83 (0.56)	3.57 (0.62)
*n* ^b^	108	111	121	229	111
Concerns of intervening (*N*^a^ = 319)	3.39 (0.60)	3.61 (0.70)	3.80 (0.64)	3.67 (0.70)	3.47 (0.59)
*n* ^b^	102	107	110	217	102

*Note.* Standard deviations in parentheses.

a Total number of participants in each variable measured.

b Number of participants per condition in each variable.

#### Testing Hypothesis 1

A 3 × 2 between groups factorial analysis of variance (ANOVA) was used to explore the effect of relationship to the victim (friend, colleague or stranger) and gender (female or male) on willingness to intervene in CC. It was hypothesized that friends and females would report the highest willingness to intervene, followed by colleagues, then strangers and males would report the least willingness to intervene. The assumption of homogeneity was analyzed using Levene’s test, which returned a non-significant value at the .01 critical alpha level. Homogeneity was further examined using a variance ratio assessment (*vr* = 1.26), which deemed the ANOVA robust to the small differences in group variance (Blanca et al., 2018).

The ANOVA found a significant main effect for condition, which explained the most variance in WTIS scores, despite having a small effect size, *F*(2, 338) = 37.030, *p* < .001 (η_*p*_^2^ = .18). A significant main effect for gender was also found, *F*(1, 339) = 14.479, *p* < .001, with a very small effect size (η_*p*_^2^ = .04). The interaction between the IV’s was non-significant, *F*(2, 338) = 1.188, *p* = .306 (*η*_p_^2^ = .01).

The group-level differences for condition IV were explored via post hoc tests using a Bonferroni correction to control for family-wise Type 1 error (Tabachnick & Fidell, 2013). Significant differences were found across all of the conditions, whereby friends (*M* = 4.06) demonstrated higher willingness to intervene than colleagues (*M* = 3.79, *p* < .001, 99% confidence interval [CI] [0.06, 0.47]) and strangers (*M* = 3.42, *p* < .001, 99% CI [0.43, 0.84]). Colleagues reported an intermediate willingness to intervene and were significantly different from strangers, who reported the least willingness to intervene (*p* < .001, 99% CI [0.17, 0.57]).

The significant gender difference was investigated via the means (see Table 1), and found that females (*M* = 3.83) reported higher willingness to intervene than males (*M* = 3.57).

#### Testing Hypothesis 2

A 3 × 2 between groups factorial ANOVA was used to explore the effect of relationship to the victim (friend, colleague or stranger) and gender (female or male) on concerns about intervening in CC. It was hypothesized that strangers and females would report the highest concerns, followed by colleagues, and then friends and males would report the least concerns. Descriptive statistics are available in Table 1. Homogeneity was assessed using Levene’s test, which was non-significant, and a variance ratio assessment (*vr* = 1.26). Based on these findings, the assumptions of normality and homogeneity were satisfied (Blanca et al., 2018).

The ANOVA results showed the overall model was significant, *F*(5, 314) = 6.191, *p* < .001 and explained 9% of the variation in CTIS scores (η_*p*_^2^ = .09). A significant, yet small, main effect for the conditions explained the most variance in CTIS scores, *F*(2, 317) = 8.282, *p* < .001, η_*p*_^2^ = .05. Additionally, a significant main effect for gender was also found, *F*(1, 318) = 7.190, *p* = 0.008, with an extremely small effect size, η_*p*_^2^ = .02. The interaction between condition and gender was non-significant, *F*(2, 317) = 1.279, *p* = .280, η_*p*_^2^ = .01 (Pallant, 2020).

Simple effects post hoc analyses using a Bonferroni correction were used to explore the group-level differences across the conditions (Tabachnick & Fidell, 2013). The difference between friends (*M* = 3.39) and strangers (*M* = 3.80) was the only significant effect, whereby friends reported significantly lower concerns of intervening than strangers (*p* ≤ .001, 99% CI [−0.67, −0.14]).

Significant gender differences were investigated via the means. The results suggested that females (*M* = 3.67) reported higher concerns about intervening than males (*M* = 3.47).

### Exploring Concerns About Intervening

The third aim was to understand which concerns about intervening in CC are most highly reported. Table 2 provides a summary of the responses.

**Table 2.** Mean and Standard Deviations for Concerns About Intervening in CC Across Relationship Conditions (*N* = 319).

Concern	Condition			Total
	Friend	Colleague	Stranger	
I would be concerned about the risk of physical harm to myself	2.94 (1.38)	3.24 (1.20)	3.72 (1.20)	3.31 (1.30)
I would be concerned about the risk of physical damage to my property	2.69 (1.36)	3.18 (1.19)	3.48 (1.27)	3.13 (1.31)
I would be concerned about creating harm for any other people involved	4.16 (0.97)	4.17 (0.92)	4.21 (0.89)	4.18 (0.92)
I would be concerned I don’t know the situation well enough to get involved	3.48 (1.09)	3.54 (1.14)	3.98 (1.08)	3.67 (1.12)
I would be concerned that I could make the situation worse for the victim	3.19 (1.12)	3.38 (1.15)	4.51 (0.74)	3.71 (1.17)
I would be concerned that if I got involved, my actions wouldn’t change anything	4.14 (0.86)	4.11 (1.08)	3.99 (0.99)	4.08 (0.98)
Before I step in, I would require more information about the situation to ensure that I hadn’t misinterpreted it	3.13 (1.13)	3.65 (1.10)	3.62 (1.35)	3.53 (1.20)
I believe this is a private matter between the couple and I don’t think I should get involved	3.24 (1.31)	3.57 (1.25)	2.85 (1.24)	3.21 (1.30)

*Note*. Standard deviations in parentheses.

The results indicated that the risk of creating harm to others involved was the most highly reported concern, with strangers reporting the highest concern and friends and colleagues reporting a similar, low level of concern. Concern that one’s intervention wouldn’t change anything was the second highest concern, with friends reporting the most concerned, followed by colleagues and then strangers. Respectively, the third and fourth highest concerns were making the situation worse for the victim and that one did not know the situation well enough to get involved. Participants were least concerned about the risk of physical damage to their property, with friends reporting the lowest level of concern.

## Discussion

To the best of our knowledge, this is the first study to investigate relational distance to a victim and concerns for bystander intervention in CC situations. The aim was to examine whether relationship between the victim and gender influenced bystanders’ willingness to intervene in CC situations. A further aim was to explore the types of concerns people were the most worried about. The results showed that victims’ friends were the most willing to intervene, while strangers reported the highest perceived concerns about intervening. In terms of gender, females indicated more willingness and concerns about intervening than males. The study also explored eight different concerns about intervening, finding that the risk of creating harm to others involved was the greatest concern for bystanders. The lowest-rated concern was the risk of physical damage to the bystander’s property.

### Willingness to Intervene

Hypothesis one predicted that both relationship to the victim and the bystanders’ gender would significantly influence willingness to intervene. In regard to the effect of relationships, it was expected that friends would report higher willingness to intervene than colleagues and colleagues more than strangers. The first part of this hypothesis was supported, indicating that the closer the relationship to the victim, the more likely the bystander is to intervene (Latané & Darley, 1970). These results are consistent with the existing literature on friendship norms (i.e., Argyle & Henderson, 1984; Essock-Vitale & Mcguire, 1980), which has proposed that the reciprocal nature of friendships facilitates the expectation that a friend should intervene in an emergency.

In regard to the bystanders’ gender, the second part of hypothesis one predicted that females would report higher willingness to intervene than males; this was supported in that females were more willing to say they would intervene. This finding is consistent with the literature (e.g., Smith et al., 2019; Yule & Grych, 2020). While this study did not measure tolerance for violence, males’ reduced willingness to intervene could suggest that men have a heightened threshold when it comes to recognizing the need for CC intervention, subsequently inhibiting their progression through the BIM steps (Yule & Grych, 2020); future research should explore this further.

### Concerns About Intervening

Hypothesis two predicted that relationship with the victim would significantly influence the concerns about intervening, whereby strangers would report the highest concerns, followed by colleagues and then friends. This hypothesis was partially supported in that strangers reported significantly higher concern than friends. This finding is akin to existing literature relating to the bystander calculus model (Piliavin et al., 1981), which proposed friends’ better understanding of the situation and increased perceived severity may reduce concerns about intervening (Banyard et al., 2021; Bennett & Banyard, 2016; Levine et al., 2005; Krieger et al., 2017; Palmer et al., 2018).

Unexpectedly, the difference between colleagues and friends was not significant and was not consistent with the existing bystander calculus literature (Banyard et al., 2021). However, the lack of difference between friends and acquaintances might be explained in terms of workplace friendships (Popper, 2020; Zarankin & Kunkel, 2019). In this study we looked at work colleagues, and as people spend a significant and regular amount of time with their colleagues, it is possible that the reciprocal nature of workplace relations acts in the same way as friendship norms (Popper, 2020), with many people considering their work colleagues as friends. For example, a large-scale survey of 15 million individuals from around the world found that approximately 30% of the survey respondents had a “best friend” whom they worked with, and more participants indicating that they had friends at work whom they did not consider best friends (Rath & Harter, 2010). As the colleague vignette was based on a 2-year long working relationship, this potentially acted as a confounding variable and future research may wish to replicate this study using a non-work acquaintance.

Similarly, the difference between colleagues and strangers was not significant. This finding could also be described in terms of work relationships (Zarankin & Kunkel, 2019). For example, it is likely that individuals may be more cautious about intervening if the colleague was their boss rather than an equal or someone under their own supervision. In particular, the relationship between a worker and their boss is characterized by a power imbalance (Dundon et al., 2017), whereby a boss has the ability to bring about additional consequences for the bystander if the intervention is misinterpreted, such as firing the employee. As the vignette did not describe the type of working relationship, it is possible that the participants perceptions of the concerns about intervening varied depending on the type of work relationship that they envisioned. Future research may benefit from using a less complex acquaintance relationship, such as a familiar stranger.

Hypothesis 2 also predicted that the bystanders’ gender would significantly influence their concerns about intervening, whereby females would report higher perceived concern than males. The research findings significantly supported this hypothesis, indicating that females may experience additional barriers to intervening in CC, possibly due to the potential for the escalation to physical aggression (Eagly & Steffen, 1986).

Interestingly, although the findings supported that females would have higher concerns, the results deviated from the assumptions of the bystander calculus model. In particular, the bystander calculus model suggested that if females report higher concerns than males, they would also be less willing to intervene (and vice versa for males) (Dovidio et al., 1991). A potential explanation for this discrepancy is that females reduced tolerance for violence and increased risk of IPV/CC victimization may be interpreted as rewards of intervening (AIHW, 2022; Fourie et al., 2017), thus outweighing the potential concerns (Dovidio et al., 1991). However, further qualitative research is required to gain a better understanding of the gender differences in the concerns and rewards of intervening in CC and whether this predicts willingness to intervene. This could raise important questions about the predictive and construct validity of the bystander calculus model.

### Exploratory Analysis of the Concerns About Intervening

The exploratory analysis exposed valuable information regarding bystanders’ concerns about intervening in CC. The greatest concerns in order were: (1) creating harm for other people involved; (2) if I got involved, my actions wouldn’t change anything; (3) I could make the situation worse for the victim; and (4) I don’t know the situation well enough to get involved. These findings appear to be consistent with the IPV literature, including Weitzman et al.’s (2020) findings, whereby the risk of harm was also the largest reported. Furthermore, the second and third largest concerns suggest that participants lacked self-efficacy concerning their ability to implement an effective intervention. The fourth largest concern was reported mostly by strangers. This is consistent with the literature on attributions of responsibility, which suggests that strangers may be more likely to ignore or delegate the intervention responsibility than friends, who typically take personal responsibility (Chabot et al., 2009; Palmer et al., 2018). While these findings demonstrate important considerations for bystander intervention program content, further statistical analysis is required to establish any significant differences in these scores and to better understand any gender differences.

### Application of the Results for Bystander Intervention Program Development

The findings from this study have implications for the development of bystander intervention programs and campaigns. First, the participants appeared to recognize a need for intervention; however, the reduced beliefs in their ability to intervene successfully and attributions of responsibility, as found in the exploratory question analysis, are important considerations for guiding the content of intervention programs. Based on these findings, program content can teach techniques or provide participants with information sources (i.e., helplines) that target these concerns and build bystanders’ confidence in their ability to successfully support victims of CC.

Secondly, friends of CC victims and females may be ideal targets for intervention programs. As evidenced in their willingness to intervene scores, friends and females may be more likely than others to recognize CC and take intervention responsibility. Additionally, the existing literature suggests that victims may be most receptive to interventions made by friends (Casey et al., 2016). Bystander intervention programs may benefit from emphasizing the importance of friends and females in CC interventions, as well as addressing the common concerns reported by these groups and providing them with the appropriate skills to increase their self-efficacy and overall likelihood of intervening.

Ideally, bystander intervention programs could address females’ higher perceived concerns about intervening. Eagly and Steffen (1986) found the potential for physical harm or similar consequences for victims and interveners was a common concern for females. Although this finding aligns with the current study results, further research may be required to gain more insight into the specific concerns reported by female bystanders in CC.

Additionally, CC is not just a female issue and effective intervention programs need to promote males’ awareness and willingness to intervene. Our findings suggest that programs must aim to challenge any deeply entrenched social norms suggesting that IPV/CC is only a women’s issue. In doing so, the broader community awareness would likely strengthen the efficacy of intervention programs. However, of note to the developers of intervention programs, there may be additional safety concerns for males intervening with male perpetrators of CC (Towns & Terry, 2014). As CC commonly forms part of men’s domestic violence against women, it is likely that there is also a “credible threat” of physical harm toward the intervener (Katz, 2022). For example, some death reviews have found that bystanders have been killed whilst intervening in domestic violence events (Department of Justice and Attorney-General, 2019). Therefore, interventions using bystander approaches in the context of CC and violence against women should carefully consider the likely consequences and risk of harm for male bystanders.

Finally, as bystander intervention programs are limited in their outreach (Mazerolle et al., 2019), an alternative method to increasing CC interventions could be through large-scale campaigns targeting the broader community. Similar to the Australian “R U OK? Day” and “Stop It at the Start” campaigns, this type of movement could specifically focus on increasing awareness of CC and provide the community with skills to intervene. The campaign could incorporate the findings from this study, by promoting interventions through friends of victims, and addressing the commonly reported concerns about intervening.

### Limitations and Future Research Directions

One limitation of this study is that although many of the findings were significant, the effect sizes were small, indicating that other variables may explain a larger proportion of the variance in bystander’s willingness and concerns about intervening in CC. The role of empathy and age may provide explanations for more of the variance in willingness. For example, Katz et al. (2015) found that victims’ friends were more likely than strangers to engage in empathetic interventions. Further, Franklin et al. (2017) found that the bystanders’ age significantly influenced willingness to intervene in SA, whereby older bystanders displayed more willingness than younger people. However, the current research regarding the effect of age on bystander behaviors appears inconclusive, with other studies finding that age has the opposite, or no effect on intervention responses. Future research may benefit from investigating the influence of empathy toward victims of CC and age of bystanders in willingness to intervene in CC.

Second, the study would have benefitted from investigating the effect of tolerance for violence on willingness to judge CC situations as (a) abusive and (b) a situation warranting intervention. Future research could include attitudinal scales measuring tolerance for violence and acceptance of CC. This would assist in gaining deeper insight into bystander behaviors in CC interventions and would further guide the content development of intervention programs.

Given the small effect sizes found in this study, it is important to note that self-report survey research may not always translate to actual behavior. In particular, this type of analogue study where participants are asked to report on hypothetical situations about intervening in CC may not reflect how they would truly behave in these situations, especially when friends or colleagues are involved. However, understanding the theoretical concerns could help to mitigate the practical concerns, when or if such a situation arises. Future research would benefit from looking further into the relationship between lived experiences around willingness to intervene and concerns before and after intervening in CC situations.

Future research could recruit participants from a wider variety of sources and use a more equal balance of genders, including LGBTQI+ and culturally diverse participants. This will help to promote the generalizability of findings more widely. Additionally, given the lack of CC research, differences in bystander responses to CC in LGBTQI+ and cross-cultural relationships are yet to be explored in detail.

Finally, given the unexpected findings in relation to colleagues’ concerns about intervening, future research could also investigate the effects of differing work relationships on bystander interventions in CC. For example, the differences between intervening with a senior, equal, and subordinate could be explored. This would be useful in understanding the role of power differentials as potential barriers to intervention.

## Conclusion

This study has contributed to the growing CC literature and demonstrates strong evidence for the potential for bystanders to intervene and potentially reduce the consequences of CC around the world. The results suggest that a bystanders’ relationship to the victim and gender can influence their intervention responses to CC, with friends of victims and females being the most willing to intervene, despite females reporting the highest concerns to intervening. The research regarding bystanders’ concerns about intervening has important implications for ensuring the content of intervention programs and public awareness campaigns is evidence-based.

## Appendix A (Vignette and Instructions as Provided to Participants)

In this study, we will be asking you to read a FICTIONAL short story that describes a conversation between a couple who are in a relationship. One member of this couple is [a friend/someone you work with/you have never met the couple]. We would be grateful if you could read the story carefully as we would like to ask some questions that are related to it.

In this study, we are really interested in what people think about this situation. Is this a private matter that people should stay out of? Or is this a situation you feel you would get involved in some way? Would you have any concerns about getting involved?

### Story

Imagine you are at your local supermarket on a busy Saturday morning, and you notice [your friend/your work colleague/a couple] in the aisle next to you. As you see [your friend/your colleague/Partner A] go to pick up a block of chocolate, their partner takes the chocolate out of their hand and raises their voice, saying “Why would you spend our money on that stuff, you’re already fat like your mother. This is why I’m not letting you see her, she’s a bad influence.”

You then find yourself a few spots behind them in the checkout line but [your friend/your colleague/Partner A] seems distracted. However, you can still hear part of the ongoing conversation. You listen as [your friend’s partner/your colleague’s partner/Partner B] tells them “We don’t need all this expensive brand stuff each week, if you were more careful with money I wouldn’t’ have to control the bank account. I saw how much money you spent at the café on cakes the other day, I can’t believe I’m with such a slob.” [Your friend/your colleague/Partner A] looks visibly uncomfortable.

As you are walking through the car park, you pass them while they are loading their groceries into the car. [Your friend’s partner/your colleagues’ partner/Partner B] still seems frustrated, and you hear them say, “No you can’t see your friends for coffee next week, you will all just snack again.” [Your friend/your colleague/Partner A] asks why and their partner replies, “I feel like I have to keep tabs on you when you go out with them, snacking and blowing our money. That’s why I have to track your phone, none of you can be trusted.” You hear them continue as they get into the car, saying “If you weren’t so ignorant and useless, I wouldn’t have to do any of this.”
